# Transcriptomic Insights into Host Metabolism and Immunity Changes after Parasitization by *Leptopilina myrica*

**DOI:** 10.3390/insects15050352

**Published:** 2024-05-14

**Authors:** Junwei Zhang, Jieyu Shan, Wenqi Shi, Ting Feng, Yifeng Sheng, Zixuan Xu, Zhi Dong, Jianhua Huang, Jiani Chen

**Affiliations:** 1 Institute of Insect Sciences, Ministry of Agriculture Key Lab of Molecular Biology of Crop Pathogens and Insect Pests, Zhejiang University, Hangzhou 310058, China; junweizhang@zju.edu.cn (J.Z.); 22316096@zju.edu.cn (J.S.); wenqishi@zju.edu.cn (W.S.); tingfeng@zju.edu.cn (T.F.); yfsheng@zju.edu.cn (Y.S.); zixuan.xu@zju.edu.cn (Z.X.); dongz@zju.edu.cn (Z.D.); jhhuang@zju.edu.cn (J.H.); 2 Key Laboratory of Biology of Crop Pathogens and Insects of Zhejiang Province, Zhejiang University, Hangzhou 310058, China

**Keywords:** parasitoid wasp, *Leptopilina myrica*, transcriptome, metabolism, immunity

## Abstract

**Simple Summary:**

The intricate coevolution between parasitoids and their hosts has long been a hot research topic. Parasitoids usually manipulate the host’s metabolism and immunity to favor the development of their offspring. In this study, we employed RNA-sequencing (RNA-seq) analysis to explore the mechanisms of the manipulation strategy of *Leptopilina myrica* on its host *Drosophila melanogaster*. A total of 445 differentially expressed genes (DEGs) were identified in host larvae at 48 h post parasitization. Among them, a large proportion of DEGs plays essential roles in host nutrition metabolism and immunity. Furthermore, the reliability of our RNA-seq data was confirmed through a qRT-PCR analysis. Our findings help to elucidate the potential mechanism underlying wasp parasitization and provide insights into their applications in biological control and integrated pest management in agriculture.

**Abstract:**

Parasitoids commonly manipulate their host’s metabolism and immunity to facilitate their offspring survival, but the mechanisms remain poorly understood. Here, we deconstructed the manipulation strategy of a newly discovered parasitoid wasp, *L. myrica*, which parasitizes *D. melanogaster*. Using RNA-seq, we analyzed transcriptomes of *L. myrica*-parasitized and non-parasitized *Drosophila* host larvae. A total of 22.29 Gb and 23.85 Gb of clean reads were obtained from the two samples, respectively, and differential expression analysis identified 445 DEGs. Of them, 304 genes were upregulated and 141 genes were downregulated in parasitized hosts compared with non-parasitized larvae. Based on the functional annotations in the Gene Ontology (GO) and Kyoto Encyclopedia of Genes and Genomes (KEGG) databases, we found that the genes involved in host nutrition metabolism were significantly upregulated, particularly in carbohydrate, amino acid, and lipid metabolism. We also identified 30 other metabolism-related DEGs, including hexokinase, fatty acid synthase, and UDP-glycosyltransferase (Ugt) genes. We observed that five Bomanin genes (Boms) and six antimicrobial peptides (AMPs) were upregulated. Moreover, a qRT-PCR analysis of 12 randomly selected DEGs confirmed the reproducibility and accuracy of the RNA-seq data. Our results provide a comprehensive transcriptomic analysis of how *L. myrica* manipulates its host, laying a solid foundation for studies on the regulatory mechanisms employed by parasitoid wasps in their hosts.

## 1. Introduction

Insects represent the most diverse and populous animal group in nature, a testament to their extraordinary capacity for evolution and adaption in varied environments. Among the Hymenoptera insects, parasitoid wasps have emerged as a particularly notable group because of their parasitic characteristics, and encompass an estimated 150,000 to 600,000 species [1]. These wasps are well-known as natural enemies of agricultural pests and are widely utilized in pest control [2,3,4]. The knowledge on parasitoid wasps have seen a substantial advancement over the last decades, enhancing our understanding of their ecological and agricultural importance [5].

Parasitoid wasps are broadly categorized as endoparasitoids and ectoparasitoids [1]. The former lay eggs inside hosts, while the latter deposit eggs on the host body surface [5,6]. Since the host nutrition and quality directly determine the fitness correlates of offspring wasps, both endoparasitoids and ectoparasitoids possess the ability to manipulate their host’s nutrition metabolism and immunity, facilitating the development of their offspring [7,8,9,10,11,12]. The parasitoid wasps utilize various factors to accomplish this manipulation, such as venom, teratocytes, larval secretions, polydnaviruses (PDVs), and virus-like particles (VLPs) [13,14,15,16,17,18,19,20,21]. For instance, a venom protein of the parasitoid wasp *Pachycrepoideus vindemiae*, PvG6PDH, has been reported to inhibit glucose-6-phosphate metabolism in its *Drosophila* host, thereby contributing to the effectiveness of parasitism [22]. The presence of dipeptidyl peptidase IV (DPPIV) in the venom of *Scleroderma guani* is capable of manipulating lipid synthesis in its host *Tenebrio molitor* [23]. *Chelonus inanitus* elevates the concentrations of free sugars in the host hemolymph and glycogen in the whole host body by injecting PDV particles, thereby ensuring the successful development of its larvae [24]. The venom of *Pteromalus puparum* has been shown to enhance the levels of soluble proteins in the hemolymph of *Pieris rapae* pupae [25]. The venom protein *Lar* of *L. heterotoma* helps to lyse host lymph glands to damage the host’s immune responses, while the venom protein Warm of *L. boulardi* helps to secure its eggs to the gut, thereby circumventing the host’s immune defenses [26]. *Cotesia vestalis* injects PDV particles into its hosts, causing apoptosis of host hemocytes and increasing the susceptibility of the hosts to bacterial infections [27]. The teratocytes of *C. flavipes* produce ICK peptides that suppress the host’s cellular immunity [28]. However, comprehensive analyses of how parasitoids manipulate their hosts are largely lacking.

The parasitoid wasps in the *Leptopilina* genus provide excellent models for studying parasitoid–host interactions [5,26,29]. *L. myrica* is a newly discovered larval-pupal parasitoid wasp of *Leptopilina*, which parasitizes 2nd instar *D. melanogaster* larvae. In this study, we employed RNA sequencing to identify differentially expressed genes (DEGs), allowing for a comparative analysis of the changes between parasitized and non-parasitized larvae. Our study will deepen our understanding of the intricate ways in which parasitoids regulate host physiological processes.

## 2. Materials and Methods

### 2.1. Insects

The parasitoid *L. myrica* was collected from Taizhou (28.65° N, 121.16° E), Zhejiang, China, in April 2021, and then maintained on the *D. melanogaster w^1118^* strain as a regular host at 25 °C and 50% relative humidity under a 16 h light and 8 h dark photoperiod. The *D. melanogaster w^1118^* hosts were fed with standard cornmeal/molasses/agar medium in 6-ounce, square bottom, plastic fly bottles, and the adult wasps were raised on apple juice agar medium (27 g agar, 33 g brown sugar, and 330 mL pure apple juice in 1000 mL diluted water) until exposure to hosts [26].

### 2.2. Samples Collection

Approximately 200 mated *Drosophila* females were allowed to lay eggs on medium within a plastic fly bottle for 1 h and then removed from the bottle. After 60 h, half of the medium with 2nd instar host larvae were transferred to another empty bottle to serve as the control group, while the remaining hosts were used to be parasitized by well-mated *L. myrica* females at a wasp/host ratio of ~1:10 for 3 h. Given that the offspring of *L. myrica* fully hatched into larvae within 48 h post parasitization, and that most parasitized *Drosophila* larvae exhibited pronounced melanization encapsulation at this time point (Figure S1)—an indicator of significant alterations in host immunity—we selected both parasitized and non-parasitized *Drosophila* larvae for comparison at 48 h post parasitization. Therefore, 40 hosts with melanotic capsules at 48 h post parasitization, *L. myrica*-parasitized and non-parasitized larvae at the same age from the control group were collected into tubes containing 500 μL of RNA-easy Isolation Reagent (R701-02-AA, Vazyme, Nanjing, China). The collected samples were immediately frozen in liquid nitrogen and stored at −80 °C until further use.

### 2.3. RNA Extraction and Illumina Sequencing

Total RNA was extracted using the FastPure Cell/Tissue Total RNA Isolation Kit-BOX2 (Cat.RC101-01, Vazyme, Nanjing, China) following the manufacturer’s instructions. The quality and quantity of the total RNA were detected using a NanoDrop 2000 (Thermo scientific, Waltham, MA, USA) and Agilent Bioanalyzer 2100/4200 (Agilent Technologies, Santa Clara, CA, USA), respectively. RNA samples then were used for library preparation. Briefly, mRNA was purified from the total RNA using oligo (dT) magnetic beads and fragmented into 300–350 bp fragments. First-strand cDNA was synthesized using random hexamer primers, followed by second-strand cDNA synthesis using DNA polymerase I and RNase H. The resulting double-stranded cDNA was subjected to end repair, phosphorylation, and ligation with Illumina paired-end sequencing adapters. The libraries were then enriched by PCR amplification and purified with an Illumina NovaSeq 6000 platform according to the manufacturer’s protocol (Berry Genomics Co. Ltd., Beijing, China).

All raw sequence data were filtered to ensure the quality and reliability. Raw FASTQ data were processed in house using scripts to obtain clean reads. Reads containing adapters, more than 3 N, or more than 20% nucleotides with a Qphred ≤5 were discarded. Additionally, the Q20, Q30, and GC content were analyzed, and the clean data were mapped to the SILVA database to remove rRNA. All subsequent transcriptome analyses were performed on the clean data.

### 2.4. Differential Gene Expression Analysis and Functional Annotation

The expression levels of unigenes in parasitized and non-parasitized larvae were obtained using the fragments per kilobase of transcript per million mapped reads (FPKM) method [30]. EdgeR was performed for the differential expression analysis between the different samples [31]. The Benjamini and Hochberg’s approach were used to adjust the resulting *p*-values to control the false discovery rate. Genes with | log2 (fold change) | > 1 and *q*-value < 0.05 were considered to be differentially expressed and were identified as DEGs. GO and KEGG enrichment analyses of the differentially expressed gene sets were implemented using the topG (http://www.bioconductor.org/packages/release/bioc/html/topGO.html, accessed on 9 August 2022) and KOBAS packages, respectively [32]. GO terms with a *p*-value < 0.05 and pathways with a *p*-value < 0.05 were considered to be significantly enriched.

### 2.5. Quantitative Real-Time PCR (qRT-PCR) Validation

Extracted total RNA from parasitized and non-parasitized larvae was reverse transcribed into cDNA using HiScript III RT SuperMix for qPCR (Vazyme, Cat#R223-01) according to the manufacturer’s protocol. qRT-PCR was performed in the QuantStudio3 Real-Time PCR System (Thermo Fisher Scientific) using the ChamQ SYBR qPCR Master Mix Kit (Cat#Q311-02, Vazyme, Nanjing, China) to validate the results from the transcriptome data. The primers used to amplify100–300 bp fragments of each PCR product are listed in Table S1. The qPCRs were performed using the following conditions: 30 s at 95 °C, followed by 40 cycles of a three-step PCR for 10 s: 95 °C, 20 s at 55 °C, and 20 s at 72 °C. Three biological replicates were performed for this assay. The RNA levels of target genes were normalized to *actin 5C* mRNA of *D. melanogaster*, and their relative concentrations in parasitized hosts were compared to those in non-parasitized larvae using the 2^-ΔΔCt^ method [33]. Statistical analyses were performed using GraphPad Prism 9.0 software (GraphPad, San Diego, CA, USA) and the data were analyzed for statistical significance using unpaired two-tailed Student’s *t*-tests. Pearson’s correlation method was used to assess the association between the qRT-PCR and RNA-seq results, and the FPKM results 48 h post *L. myrica* parasitization detected by RNA-seq were plotted against the qRT-PCR data.

## 3. Results

### 3.1. Transcriptomes of the Host Larvae after Parasitism by L. myrica

**Figure 1:**
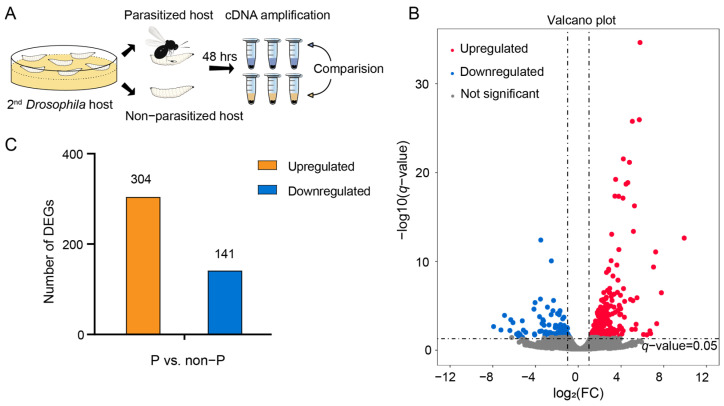
Experimental design and identification of DEGs between the parasitized and non-parasitized groups. (**A**) Experimental design and comparisons employed in this study. Transcriptomes were generated from Drosophila host larvae at 48 h following parasitization by *L. myrica*, and non-parasitized individuals at the same developmental stages served as the control. (**B**) Volcano plot of the 16,941 unigenes; each point in the volcano diagram represents one unigene, and only those with | log2 (FC) | > 1 and a q-value < 0.05 were identified as DEGs. The red points represent the upregulated DEGs, the blue points represent the downregulated DEGs, and the gray points represent the unigenes that are not significant. (**C**) Number of DEGs identified from the parasitized (P) and non-parasitized (non-P) Drosophila host groups. The orange column represents the upregulated DEGs and the blue column represents the downregulated DEGs.

To comprehensively characterize the transcriptional response of host larvae after *L. myrica* parasitization, cDNAs were generated from samples of non-parasitized and parasitized larvae, followed by sequencing using the Illumina NovaSeq 6000 platform (Figure 1A). We obtained a total of 22.29 Gb and 23.85 Gb of clean reads from the two different treatments. Three independent biological replicates were sequenced for each condition, resulting in a range of 5.95–8.25 Gb of clean bases for each non-parasitized larva sample and 6.63–8.92 Gb of clean bases per parasitized larva sample (Table 1). In our results, the GC content across the six distinct samples exhibited a range from 48.23% to 50.27%, while the rRNA ratio varied between 2.62% and 7.57%. The RNA-seq data showed good quality, as evidenced by the Q20 quality values (sequencing error rate < 1%) exceeding 96.21%, and the Q30 quality values (sequencing error rate < 0.1%) surpassing 90.49% in all six samples. Subsequent mapping of the RNA-seq reads to the *D. melanogaster* reference genome (GCA_000001215.4) revealed a high mapping efficiency, with 89.94% to 96.68% of the reads aligning to the reference genome (Table 1). Finally, a total of 16,941 unigenes were assembled across all six samples (Table S2).

### 3.2. Analysis of Differentially Expressed Genes (DEGs)

**Figure 2:**
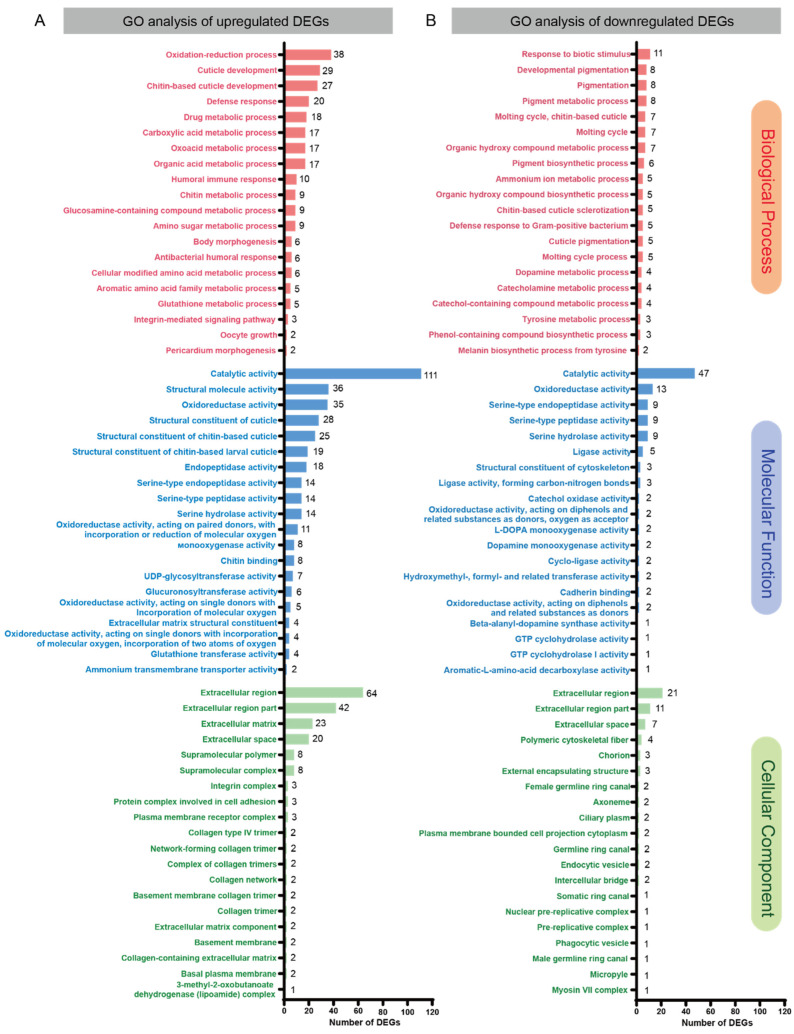
GO classification of the DEGs between parasitized and non-parasitized Drosophila larvae at 48 h after parasitization. Top 20 enriched GO classifications of annotated upregulated (**A**) and downregulated (**B**) DEGs. The distributions are summarized into three main categories: biological processes (BP), molecular functions (MF), and cellular components (CC). The *x*-axis shows the number of DEGs in each category, and the *y*-axis shows the different GO terms.

The volcano plots show a total of 445 DEGs between *L. myrica*-parasitized and non-parasitized *D. melanogaster* hosts according to the conditions of | log2 (fold change) | > 1 and a *q*-value < 0.05 (Figure 1B), including 304 upregulated genes and 141 downregulated genes (Figure 1C). All 445 DEGs are presented in Table S3. These identified DEGs were subjected to GO analysis for functional annotation across three categories: biological processes (BP), molecular functions (MF), and cellular components (CC) (Table S4). In addition, the top 20 enriched GO classifications for each category were systematically cataloged and listed (Figure 2). In the BP ontology of the GO classification, a notable enrichment was observed in the upregulated DEGs associated with the “oxidation–reduction process”, featuring 38 DEGs (Figure 2A). Concurrently, in the MF category, the most significant enrichment among the upregulated DEGs was identified in “catalytic activity”, encompassing 111 DEGs (Figure 2A). In the CC category, the upregulated DEGs were predominantly linked to the “extracellular region”, with a total of 64 DEGs (Figure 2A). Furthermore, the analysis revealed that the most downregulated GO term in the BP category was “response to biotic stimulus”, consisting of 11 DEGs (Figure 2B), while in the MF category, “catalytic activity” was the most affected, with 47 downregulated DEGs (Figure 2B). In the CC category, “extracellular region” was predominantly associated with downregulated DEGs, including 21 DEGs (Figure 2B). Collectively, these results implied an obvious change in the physiological processes of the host 48 h post parasitization.

To elucidate the intricate molecular interactions and networks influenced by *L. myrica* parasitization, we conducted a comprehensive analysis of 445 DEGs in relation to their involvement in KEGG pathways. We identified 18 KEGG pathways associated with upregulated DEGs and 5 KEGG pathways corresponding to downregulated DEGs (Table 2). Notably, within the 18 pathways that were enriched with the upregulated DEGs, a substantial majority (16/18, 88.89%) pertained to metabolic processes, primarily encompassing six classes, which included “carbohydrate metabolism”, “xenobiotic biodegradation and metabolism”, “amino acid metabolism”, “metabolism of cofactors and vitamins”, “lipid metabolism”, and “global and overview maps”. The remaining two pathways, which are not involved in metabolic processes, were categorized under environmental information processing. Interestingly, a similar pattern was found in the downregulated DEGs, where all five enriched pathways were related to metabolic processes. These encompassed four processes: “amino acid metabolism”, “carbohydrate metabolism”, “lipid metabolism”, and “metabolism of cofactors and vitamins”. The KEGG annotations provided new insights into the complex metabolic regulation in host larvae post *L. myrica* parasitization.

### 3.3. DEGs in Nutrition Metabolic Processes

**Figure 3:**
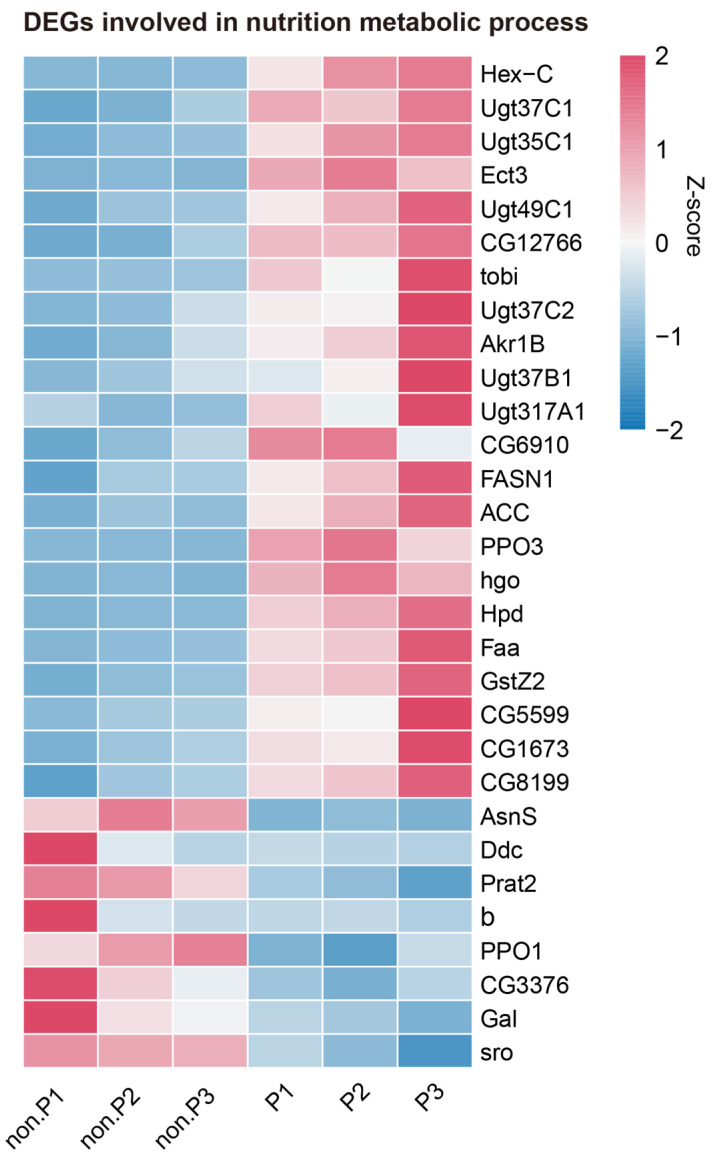
Expression profiles of DEGs involved in carbohydrate metabolism, amino acid metabolism, and lipid metabolism. Each column represents an individual parasitized or non-parasitized larva sample. The color gradient from blue to red represents low to high gene expression levels normalized using Z-score normalization. Abbreviations: non-P, non-parasitized larvae; P, parasitized host; Hex-C, hexokinase C; Ugt37C1, UDP-glycosyltransferase family 37 member C1; Ugt35C1, UDP-glycosyltransferase family 35 member C1; Ect3, ectoderm-expressed 3; Ugt49C1, UDP-glycosyltransferase family 49 member C1; tobi, target of brain insulin; Ugt37C2, UDP-glycosyltransferase family 37 member C2; Akr1B, aldo-keto reductase 1B; Ugt37B1, UDP-glycosyltransferase family 37 member B1; Ugt317A1, UDP-glycosyltransferase family 317 member A1; FASN1, fatty acid synthase 1; ACC, acetyl-CoA carboxylase; PPO3, prophenoloxidase 3; hgo, homogentisate 1,2-dioxygenase; Hpd, 4-hydroxyphenylpyruvate dioxygenase; Faa, fumarylacetoacetase; GstZ2, glutathione S transferase Z2; AsnS, asparagine synthetase; Ddc, dopa decarboxylase; Prat2, phosphoribosylamidotransferase 2; b, black; PPO1, prophenoloxidase 2; Gal, β galactosidase; sro, shroud.

Carbohydrates, along with proteins and lipids, constitute the primary classes of organic compounds in insects. Within the 16 upregulated KEGG pathways that are related to metabolism in our analysis (Table 2), a significant proportion of the pathways (9/16, 56.25%) was intricately linked to the metabolic processes of carbohydrates (KEGG: dme00053, dme00500, dme00040, dme00052, dme00640, and dme00051), amino acids (KEGG: dme00350 and dme00280), and lipids (KEGG: dme00061). Similarly, among the five metabolism-related KEGG pathways that were found to be downregulated in Table 2, a predominant proportion of the pathways (4/5, 80%) was directly implicated in the metabolic processes of carbohydrates (KEGG: dme00650), amino acids (KEGG: dme00250 and dme00350), and lipids (KEGG: dme00600). These observations indicated a notable alteration in the host’s nutrition metabolic processes due to *L. myrica* parasitization. Consequently, we identified 30 DEGs across 13 nutrition metabolism-related KEGG pathways, which comprised 22 upregulated and 8 downregulated genes, including hexokinase, UDP-glycosyltransferase (Ugt), fatty acid synthase, etc. (Figure 3). Remarkably, six Ugt genes (*Ugt37C1*, *Ugt35C1*, *Ugt49C1*, *Ugt37C2*, *Ugt37B1*, and *Ugt317A1*) showed a greater than 2.64-fold higher expression at 48 h post *L. myrica* parasitization.

### 3.4. DEGs in Immunity Processes

**Figure 4:**
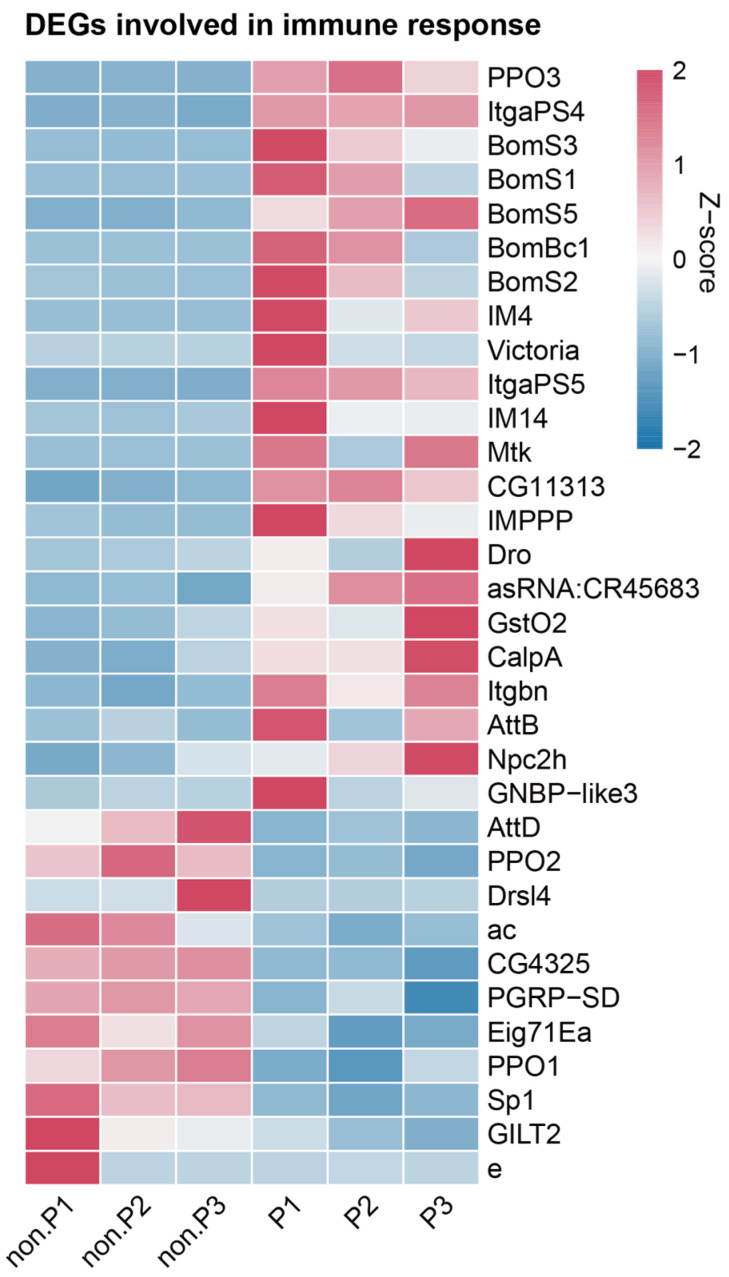
Expression profiles of DEGs involved in immune responses. Each column represents an individual parasitized or non-parasitized larva sample. The color gradient from blue to red represents low to high gene expression levels normalized using Z-score normalization. Abbreviations: non-P, non-parasitized larvae; P, parasitized host; PPO3, prophenoloxidase 3; ItgaPS4, integrin alphaPS4 subunit; BomS3, Bomanin short 3; BomS1, Bomanin short 1; BomS5, Bomanin short 5; BomBc1, Bomanin bicipital 1; BomS2, Bomanin short 2; IM4, immune-induced molecule 4; ItgaPS5, integrin alphaPS5 subunit; IM14, immune-induced molecule 14; Mtk, metchnikowin; IMPPP, Baramicin A2; Dro, drosocin; GstO2, glutathione S transferase O2; CalpA, calpain-A; Itgbn, integrin betanu subunit; AttB, attacin B; Npc2h, Niemann–Pick type C-2h; AttD, attacin D; PPO2, prophenoloxidase 2; Drsl4, drosomycin-like 4; ac, achaete; PGRP-SD, peptidoglycan recognition protein SD; Eig71Ea, ecdysone-induced gene 71Ea; PPO1, prophenoloxidase 1; GILT2, gamma-interferon-inducible lysosomal thiol reductase 2; e, ebony.

Based on the BP ontology of the GO classification, we found half of the top 20 enriched GO classifications, within both the upregulated and downregulated datasets, were directly related to metabolic processes (Figure S2, blue font). However, our analysis also showed some classifications linked to immunity processes (Figure S2, red font). Within the top 20 enriched GO terms in the BP category for upregulated genes, 4 classifications were identified as “defense response”, “antibacterial humoral response”, “integrin-mediated signaling pathway”, and “humoral immune response” (Figure S2A), which contained 20, 6, 3, and 10 DEGs, respectively (Figure 2A). On the other hand, the analysis showed 2 immune response-related classifications among the downregulated top 20 enriched GO terms, including “defense response to Gram-positive bacterium” and “response to biotic stimulus” (Figure S2B), which contained 5 and 11 DEGs, respectively (Figure 2B). We then focused on the immunity-related DEGs obtained from the six enriched GO classifications. A total of 33 DEGs were discerned through a comparative analysis between the parasitized and non-parasitized groups (Figure 4). Among them, twenty-two (22/33, 66.67%) DEGs showed a greater than 2.21-fold higher expression, whereas the remaining 11 (11/33, 33.33%) DEGs showed a greater than 0.47-fold lower expression at 48 h post *L. myrica* parasitization. Interestingly, we observed a notable increase in the expression levels of five Bomanin genes (*BomS3*, *BomS1*, *BomS5*, *BomBc1*, and *BomS2*) and six AMPs (*IM4*, *IM14*, *IMPPP*, *Mtk*, *Dro* and *AttB*) (Figure 4). Moreover, one prophenoloxidase gene (*PPO3*) was upregulated in the parasitized host larvae at 48 h, while other two prophenoloxidase genes (*PPO2* and *PPO1*) showed decreased expression after *L. myrica* parasitization.

### 3.5. Verification of DEG Expression

**Figure 5:**
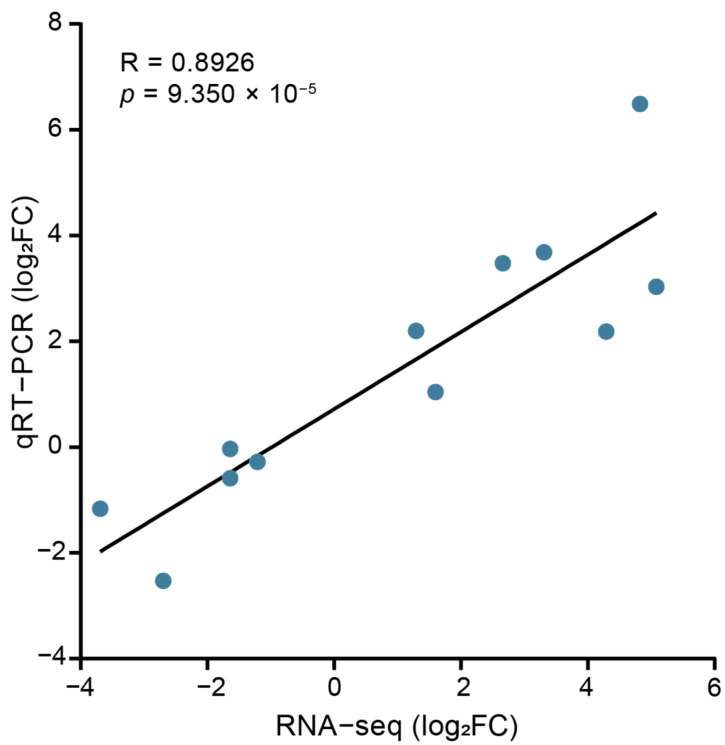
Validation of RNA-seq data using qRT-PCR. Log2(FC) in gene expression following 48 h of *L. myrica* parasitization detected by RNA-seq plotted against the qRT-PCR data. The reference line indicates a linear relationship between the qRT-PCR and RNA-seq results (Pearson correlation coefficient, R = 0.8926; *p* = 9.350 × 10^−5^).

To validate the accuracy and reproducibility of the expression patterns of the DEGs identified from our RNA-seq data, a total of 12 DEGs, namely *Tep1*, *CG8160*, *IMPPP*, *BomS3*, *FASN1*, *hpd*, *hgo*, *ac*, *PPO2*, *pav*, *hll*, and *AsnS*, were randomly selected for confirmation by qRT-PCR. Among these DEGs, four genes (*FASNone*, *hpd*, *hgo* and *AsnS*) are involved in host metabolism, and four genes (*IMPPP*, *BomS3*, *ac* and *PPO2*) are involved in host immunity. The qRT-PCR results indicated that the expression of *Tep1*, *CG8160*, *IMPPP*, *BomS3*, *FASN1*, *hpd*, and *hgo* showed a 2.06–89.70-fold increase at 48 h post *L. myrica* parasitization (Figure S3). Meanwhile, the expression levels of the other five DEGs (ac, *PPO2*, *pav*, *hll*, and *AsnS*) showed a marked reduction (0.17–0.98-fold) in the parasitized hosts in comparison with the non-parasitized larvae (Figure S3). Fold changes (FCs) in expression levels obtained from the RNA-seq and qRT-PCR data were graphically represented on a scatter plot, with the log2 (FC) values from RNA-seq plotted on the *x*-axis and values from qRT-PCR plotted on the *y*-axis (Figure 5). Furthermore, the Pearson correlation coefficient (R = 0.8926, *p* = 9.350 × 10^−5^) demonstrated a significant positive correlation between the data from the two techniques of RNA-seq and qRT-PCR.

## 4. Discussion

Almost all parasitoid wasps have a free-living lifestyle as adults; however, their offspring at the larval stage must develop in or on their hosts. This complex life cycle needs the delicate manipulation of the host’s physiological processes, especially those related to metabolism and immunity, to facilitate their own growth and development [24,34,35,36,37]. For example, *P. vindemiae* can inhibit glucose-6-phosphate metabolism in the host to facilitate its own parasitism, suggesting that alterations in host carbohydrate metabolism can significantly influence the key fitness correlates of the parasitoid [22]. *C. vestalis* stimulates a reduction in host lipid levels, benefiting the development of its wasp offspring [38]. *Meteorus pulchricornis* enhances trehalose metabolism in its host, *Spodoptera litura*, to improve the fitness of its offspring [39]. *M. pallidipes* parasitization increases the lipid content in its *S. exigua* host [40]. *P. puparum* parasitization induces the activity of α-amylases and influences the carbohydrate metabolism of its butterfly host [8]. *L. boulardi* parasitization increases the concentration of diptericin, an antibacterial peptide, helping the host to produce an effective humoral immune response to *Escherichia coli* [41]. Previous studies suggest that parasitoids typically manipulate the host’s metabolism and immunity in ways that favor the development of their offspring. These strategic alterations ensure that the parasitoid larvae have the necessary nutritional resources and a reduced risk of immunity challenges from the host, thereby enhancing their survival and successful development. Recently, we have discovered a new *Leptopilina* species, *L. myrica*. To comprehensively study the underlying mechanisms of the manipulation strategy used by *L. myrica* on its host, we compared the transcriptional profiles of *L. myrica*-parasitized and non-parasitized larvae.

A total of 445 DEGs were identified between the two different groups of larvae, comprising 304 upregulated and 141 downregulated genes (Figure 1C). The KEGG pathway analysis illuminated a significant enrichment in metabolic processes among the DEGs, with a notable upregulation of essential energy substances such as carbohydrates, amino acids, and lipids (Table 2), which may provide the essential nutrients for the development and survival of *L. myrica* offspring [34]. We also analyzed the expression profiles of DEGs related to nutrition metabolic processes (Figure 3). Among the 30 DEGs, a significant proportion (22/30, 73%) were upregulated, and 12 DEGs (*Hex-C*, *Ect3*, *CG12766*, *tobi*, *Akr1B*, *CG6910*, *Ugt37C1*, *Ugt35C1*, *Ugt49C1*, *Ugt37C2*, *Ugt37B1*, and *Ugt317A1*) are involved in carbohydrate metabolic processes. Several studies have demonstrated an increased level of sugars within parasitized hosts, which benefits the development of parasitoid juveniles [24,36]. *Hex-C* is a predominant isoform of hexokinases, and its upregulation enhanced glucose utilization and storage [42,43]. Another upregulated gene, *tobi*, is responsible for glycogen degradation [44], potentially facilitating the absorption of host glycogen by the parasitoid larvae. Importantly, all six Ugt family genes (*Ugt37C1*, *Ugt35C1*, *Ugt49C1*, *Ugt37C2*, *Ugt37B1*, and *Ugt317A1*) in host larvae were upregulated after *L. myrica* parasitization. It has been reported that Ugt genes play a crucial role in the detoxification of toxic substances encountered in food or the living environment [45,46,47]. For instance, *M. pulchricornis* utilizes Ugt genes to detoxify the phytoalexins from plants [48]. *Helicoverpa armigera* and its closely related species *H. assulta* exhibit distinct adaptations to the feeding deterrent capsaicin by employing Ugt [49]. As such, our results suggested that the increased expression of Ugts might enhance the host resistance to toxic substances in the living environment, thereby protecting *L. myrica* from external toxicity. We also screened eight upregulated DEGs (*PPO3*, *hgo*, *Hpd*, *Faa*, *GstZ2*, *CG5599*, *CG1673*, and *CG8199*) that are involved in amino acid metabolic processes. *Hpd* has been implicated in the tyrosine catabolic process, playing a pivotal role in the formation of insect epidermis [50]. Increased expression levels of *hpd* may lead to a faster cuticle maturation of parasitoid wasp larvae, thus enhancing their ability to defend against the host immune responses and improve their survival. Given that parasitoid larvae obtain all the necessary nutrients from their hosts, they directly derive lipids from their hosts, consequently diminishing their own ability for lipid synthesis [51,52,53]. This adaptation was further evidenced by the two upregulated host fatty acid synthesis genes (*FASN1* and *ACC*), supporting the perspective that parasitoids regulate the host’s lipid metabolism for their own benefit. *Sro* is important for ecdysone biosynthesis, and the downregulation of *Sro* post *L. myrica* parasitization might illustrate the influence of parasitoids on the host ecdysis process, decelerating the host’s development and providing the parasitoid with more time to absorb nutrients from the host [54,55]. Based on these results, the parasitoid wasps have evolved to manipulate the host’s nutrition metabolism, such as carbohydrate metabolism, amino acid metabolism, and lipid metabolism, to secure the successful postembryonic development of their offspring.

It is well known that the host immunity process will change in response to wasp parasitization [56,57]. Previous studies have documented alterations in host immune responses induced by parasitism from diverse species of parasitic wasps, showing a dual manipulation of host immunity [2,34,58]. In this study, we found similar results, finding that 33.33% (11/33) of the immune-related DEGs were downregulated and 66.67% (22/33) of the immune-related DEGs were upregulated. In the upregulated immune-related DEGs, there were four primary functional categories: “defense response”, “antibacterial humoral response”, “integrin-mediated signaling pathway”, and “humoral immune response”. Specifically, we found that five Boms, namely *BomS1*, *BomS2*, *BomS3*, *BomS5*, and *BomBc1*, were upregulated at 48 h post *L. myrica* parasitism (Figure 4). Previous studies on *Drosophila* hosts have reported that the expression of Boms enhances antifungal activity [59,60,61,62]. In our study, the upregulation of Boms suggests that these proteins may help enhance the host’s resistance to fungal infections, thus creating a more favorable environment for the developing parasitoid offspring. Prophenoloxidases (PPOs) play an important part in melanin formation at infection sites [63]. In some parasitic systems, such as *S. frugiperda*–*Microplitis manila*, *Pseudaletia separate*–*M. mediator*, and *P. rapae–P. puparum*, the PPOs in infected hosts were suppressed post wasp parasitization, allowing the parasitoids to overcome the host melanin-based immune defenses [34,64,65]. However, in other parasitic systems, like *Asobara tabida*–*D. melanogaster* and *A. citri*–*D. melanogaster*, PPOs were activated in parasitized host larvae [66]. Our results showed that the expression levels of three PPOs were significantly altered after parasitization. While *PPO1* and *PPO2* were suppressed, *PPO3* was upregulated. Previous studies suggested that *PPO3* alone may not be sufficient to produce adequate melanization without the help of *PPO1* and *PPO2* [63,67,68]. This could be a potential reason why *L. myrica* can successfully evade the host immune response. Simultaneously, antimicrobial peptides, including *IM4*, *IM14*, *IMPPP*, *Mtk*, *Dro*, and *AttB*, were upregulated after parasitization, indicating an increased resistance of the *Drosophila* hosts against infection by bacteria and fungi [69,70,71,72,73]. These findings thus expand our knowledge on the parasitic strategy of balancing the immune status in the parasitized hosts to benefit the parasitoid wasp larvae.

## 5. Conclusions

In summary, we performed a comparative transcriptome analysis using RNA-seq to investigate the changes in hosts post parasitization. Our findings provide novel insights into host metabolism and immunity alterations after parasitization by parasitoid wasps, which will not only broaden our knowledge on the coevolutionary adaptations in this parasitic relationship but also contribute to developing sustainable pest management strategies harnessing the power of natural enemies.

## Figures and Tables

**Table 1:** Basic summary of RNA-sequencing results.

Sample Name	Clean Reads (bp)	Clean Bases (bp)	Clean GC (%)	Clean Q20 (%)	Clean Q30 (%)	rRNA Ratio (%)	Total Mapped (%)
non-P1	39,663,576	5,949,536,400	48.95; 48.98	97.49; 96.77	93.31; 91.83	2.62	96.26
non-P2	54,988,600	8,248,290,000	49.81; 49.82	97.60; 96.98	93.46; 92.21	7.57	96.68
non-P3	53,963,624	8,094,543,600	49.26; 49.30	97.62; 97.81	93.69; 93.65	2.69	96.61
P1	59,486,256	8,922,938,400	48.23; 48.36	97.21; 96.21	92.86; 90.49	4.28	89.94
P2	55,431,152	8,314,672,800	50.27; 50.26	98.08; 97.45	94.53; 93.02	4.51	91.21
P2	44,168,656	6,625,298,400	48.79; 48.86	97.54; 97.20	93.45; 92.15	5.69	91.61

Abbreviations: non-P, non-parasitized larvae; P, parasitized host.

**Table 2:** KEGG pathways significantly enriched with DEGs identified from the parasitized and non-parasitized *Drosophila* larvae at 48 h after parasitization.

Expression Level	KEGG Pathway	Category	Class	Description	*p*-Value	Number of Genes	Ratio (%)
Upregulated	dme00053	Metabolism	Carbohydrate metabolism	Ascorbate and aldarate metabolism	7.90 × 10^−6^	7	5.69
	dme00500			Starch and sucrose metabolism	6.65 × 10^−5^	8	6.50
	dme00040			Pentose and glucuronate interconversions	1.45 × 10^−5^	8	6.50
	dme00052			Galactose metabolism	1.33 × 10^−3^	5	4.07
	dme00640			Propanoate metabolism	1.12 × 10^−2^	3	2.44
	dme00051			Fructose and mannose metabolism	2.45 × 10^−2^	3	2.44
	dme00983		Xenobiotics biodegradation and metabolism	Drug metabolism—other enzymes	1.65 × 10^−5^	8	6.50
	dme00982			Drug metabolism—cytochrome P450	6.65 × 10^−5^	8	6.50
	dme00980			Metabolism of xenobiotics by cytochrome P450	6.65 × 10^−5^	8	6.50
	dme00860		Metabolism of cofactors and vitamins	Porphyrin and chlorophyll metabolism	4.21 × 10^−4^	6	4.88
	dme00770			Pantothenate and CoA biosynthesis	3.42 × 10^−2^	2	1.63
	dme00830			Retinol metabolism	7.90 × 10^−6^	7	5.69
	dme00350		Amino acid metabolism	Tyrosine metabolism	4.46 × 10^−5^	5	4.07
	dme00280			Valine, leucine, and isoleucine degradation	3.57 × 10^−2^	3	2.44
	dme00061		Lipid metabolism	Fatty acid biosynthesis	1.88 × 10^−2^	2	1.63
	dme01100		Global and overview maps	Metabolic pathways	4.52 × 10^−4^	36	29.27
	dme02010	Environmental Information Processing	Membrane transport	ABC transporters	1.88 × 10^−2^	2	1.63
	dme04512		Signaling molecules and interaction	ECM–receptor interaction	2.60 × 10^−2^	2	1.63
	dme00250	Metabolism	Amino acid metabolism	Alanine, aspartate, and glutamate metabolism	3.60 × 10^−3^	3	27.27
Downregulated	dme00350			Tyrosine metabolism	1.02 × 10^−2^	2	18.18
	dme00650		Carbohydrate metabolism	Butanoate metabolism	1.26 × 10^−2^	2	18.18
	dme00600		Lipid metabolism	Sphingolipid metabolism	3.62 × 10^−2^	2	18.18
	dme00670		Metabolism of cofactors and vitamins	One carbon pool by folate	7.14 × 10^−3^	2	18.18

## Data Availability

The raw Illumina sequences of all six samples are available in the Sequence Read Archive (SRA) under accession PRJNA978010. All data presented in this study are available in the article and Supplementary Material.

## Supplementary Materials

The following supporting information can be downloaded at: https://www.mdpi.com/article/10.3390/insects15050352/s1, Figure S1: *L. myrica* parasitization triggers melanotic response in infected fly larvae; Figure S2: GO enrichment in BP category of the DEGs between parasitized and non-parasitized larvae; Figure S3: Validation of RNA sequencing data by qRT-PCR; Table S1: Primers used for quantitative real-time PCR (5′-3′); Table S2: The list of 16,941 unigenes assembled across all 6 samples; Table S3: The list of 445 DEGs in P vs. non-P; Table S4: GO analysis of identified DEGs.
