# Supplementary material for: Transcriptomic Insights into Host Metabolism and Immunity Changes after Parasitization by Leptopilina myrica
Source: Insects. 2024 May 14;15(5):352. doi: 10.3390/insects15050352 (PMC11122121; doi:10.3390/insects15050352)
Supplement: Supplementary file 1 [file insects-15-00352-s001.zip › Supplementary Figures.pdf]

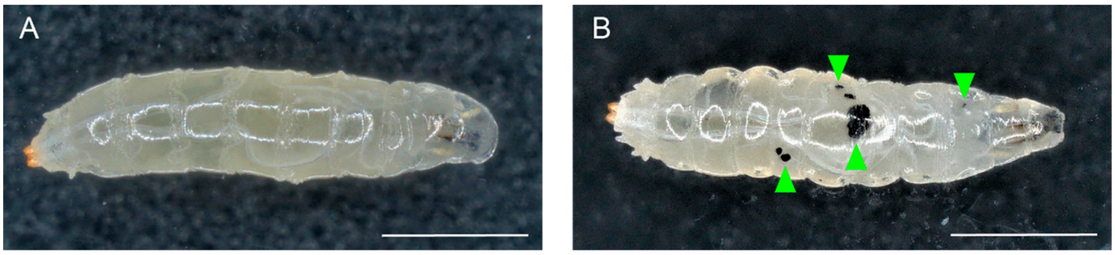

Figure S1. ***L. myrica* parasitization triggers melanotic response in infected fly larvae.**

Representative image of non-parasitized *Drosophila* third instar larvae (**A**) and those 48 h after parasitization by *L. myrica* (**B**). Green arrowheads in (**B**) represent melanotic capsule formation. Scale bars: 1 mm.

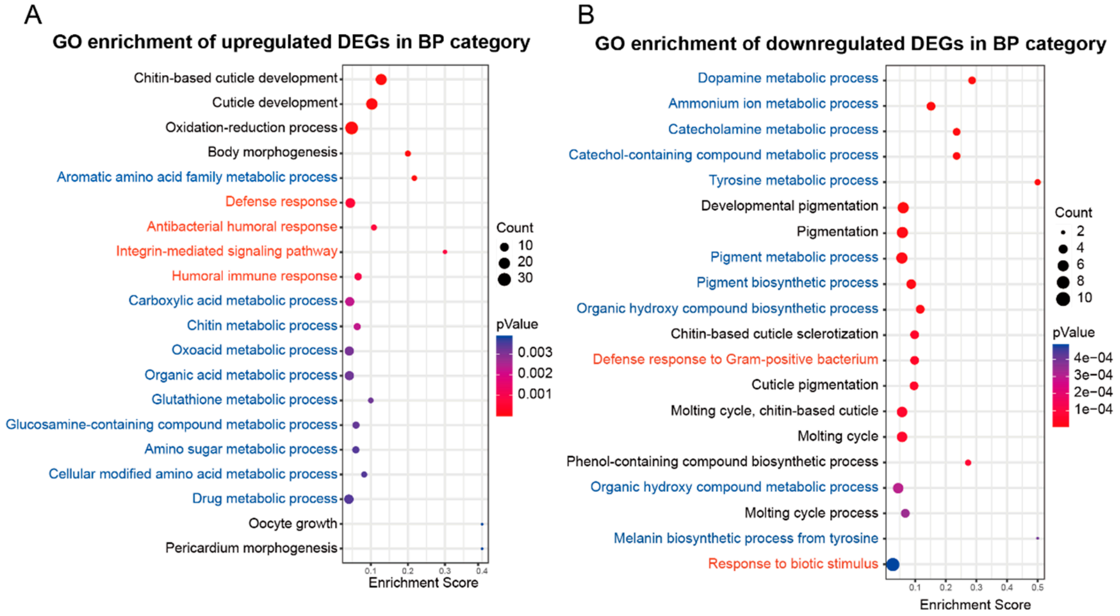

Figure S2. **GO enrichment in biological process BP category of the DEGs between the comparisons of parasitized and non-parasitized hosts.**

(**A**) Top 20 enriched GO terms in the BP category for upregulated genes. (**B**) Top 20 enriched GO terms in the BP category for downregulated genes. The color and size of the dots respectively indicated the range of the *p* value and the number of DEGs mapped to the pathways.

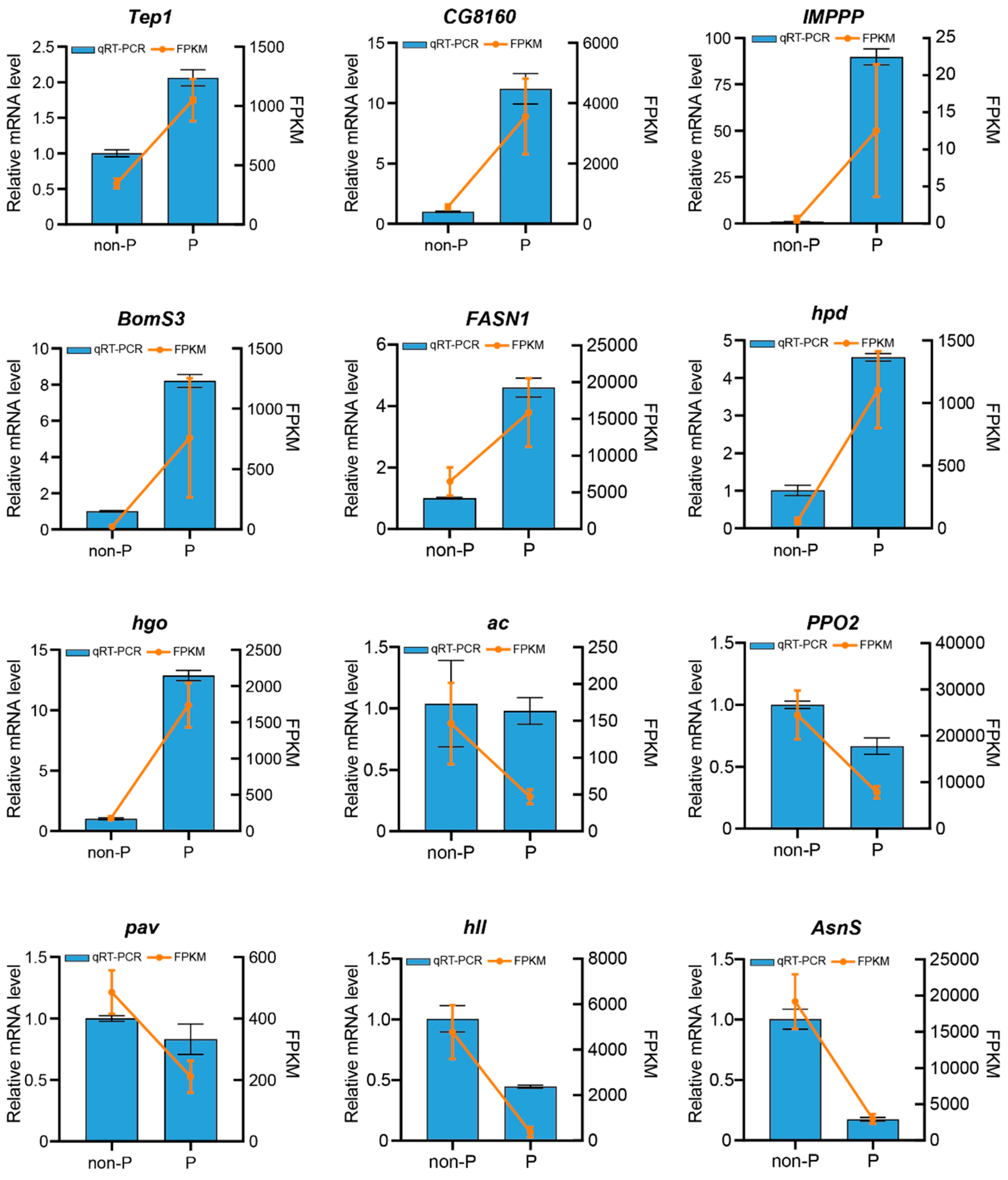

Figure S3. **Validation of RNA sequencing data by qRT-PCR.**

The blue bars indicate the relative expression levels of twelve genes by qRT-PCR. The red lines represent the expression levels obtained from the RNA-seq data using the FPKM method. The values represent the means ± SDs of three independent experiments.
